# Midbrain dopaminergic degeneration differentially modulates primary motor cortex activity and motor behavior in hemi-parkinsonian rats

**DOI:** 10.21203/rs.3.rs-4365911/v1

**Published:** 2024-05-14

**Authors:** Suelen L. Boschen, Julian Seethaler, Shaohua Wang, Wendy D. Lujan, Jodi L. Silvernail, Rickey E. Carter, Su-Youne Chang, J. Luis Lujan

**Affiliations:** Mayo Clinic; Paracelsus Medical University; National Institute of Environmental Health Sciences; Mayo Clinic; Mayo Clinic; Mayo Clinic; Mayo Clinic; Mayo Clinic

**Keywords:** Parkinson’s disease (PD), Primary motor cortex (M1), in vivo calcium imaging, neuronal calcium activity, dopaminergic transmission

## Abstract

Parkinson’s disease (PD) is marked by degeneration in the nigrostriatal dopaminergic pathway, affecting motor control via complex changes in the cortico-basal ganglia-thalamic motor network, including the primary motor cortex (M1). The modulation of M1 neuronal activity by dopaminergic inputs, particularly from the ventral tegmental area (VTA) and substantia nigra pars compacta (SNc), plays a crucial role in PD pathophysiology. This study investigates how nigrostriatal dopaminergic degeneration influences M1 neuronal activity in rats using in vivo calcium imaging. Histological analysis confirmed dopaminergic lesion severity, with high lesion level rats showing significant motor deficits. Levodopa treatment improved fine motor abilities, particularly in high lesion level rats. Analysis of M1 calcium signals based on dopaminergic lesion severity revealed distinct M1 activity patterns. Animals with low dopaminergic lesion showed increased calcium events, while high lesion level rats exhibited decreased activity, partially restored by levodopa. These findings suggest that M1 activity is more sensitive to transient fluctuations in dopaminergic transmission, rather than to chronic high or low dopaminergic signaling. This study underscores the complex interplay between dopaminergic signaling and M1 neuronal activity in PD symptoms development. Further research integrating behavioral and calcium imaging data can elucidate mechanisms underlying motor deficits and therapeutic responses in PD.

## Introduction

Parkinson’s disease (PD) is a progressive neurodegenerative disease that primarily affects the nigrostriatal dopaminergic pathway resulting in movement abnormalities, including bradykinesia, tremor, and muscular rigidity.^1–3^ The pathophysiology of PD spreads from the nigrostriatal pathway through the cortico-basal ganglia-thalamic motor network with complex changes in activity in several regions, including the primary motor cortex (M1).^4–6^

The M1 is critically important in motor learning and volitional movement control as it is responsible for initiating downstream motor activation.^7–9^ Dopaminergic inputs from the ventral tegmental area (VTA) and, to a lesser extent, from the substantia nigra *pars compacta* (SNc) directly modulate M1 neuronal activity via D1-like (D1R) and D2-like (D2R) dopaminergic receptors^10,11^ by modulating the firing rate and synchronization of M1 neurons. Indirect modulation of M1 activity is also achieved via SNc dopaminergic projections onto the direct and indirect pathways of the basal ganglia.^12–14^ In PD, the loss of SNc dopaminergic input exacerbates the indirect pathway activity while weakening the direct pathway, leading to increased inhibition of motor thalamic nuclei and decreased M1 excitation, contributing to dysfunctional motor output.^15–17^ However, functional imaging studies show that M1 activity may be either increased or decreased in both PD patients and animal models of PD.^18–26^ Similarly, conflicting results from single-unit electrophysiological recordings in non-human primate M1, show either no changes or a reduction in M1 activity after MPTP treatment.^27–29^ In contrast, studies in hemi-parkinsonian rats have shown increased synchronicity and beta-frequency oscillations between M1 and striatum.^30^ Therefore, a cohesive interpretation of the pathological changes in M1 activity in the context of pathological behaviors and therapeutic responses remains elusive.

**Figure 1:**
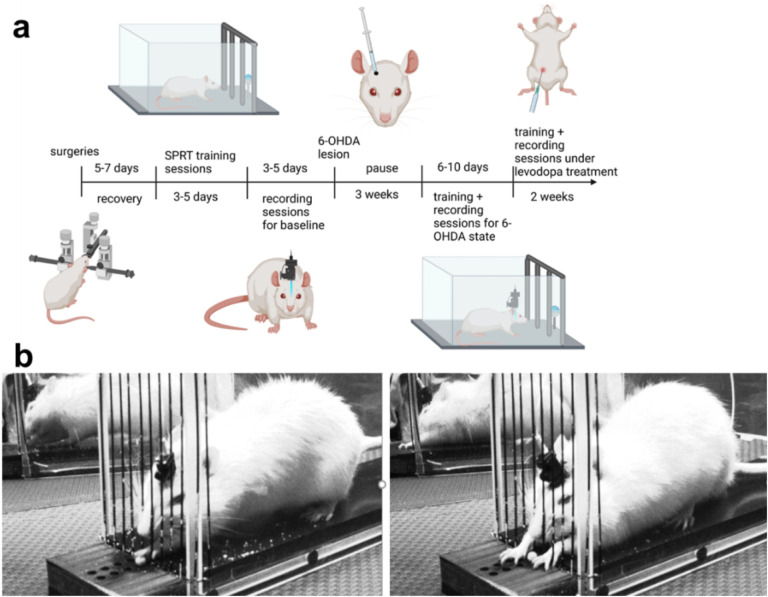
Experimental design. The experimental protocol (a) consisted of surgical procedures to inject GCaMP6f and implant a PRISM lens in the M1, implant a guide-cannula in the medial forebrain bundle (MFB), and attach a baseplate for subsequent miniature microscope connection. Following recovery, longitudinal assessments of fine motor function were performed using the single pellet reaching test (SPRT) during three conditions: normal physiological state (naive), following a unilateral intra-MFB infusion of 6-OHDA, and during the course of levodopa treatment. Once the longitudinal assessment was completed, rats were euthanized and their brains were extracted for histological analysis. (b) Representative images of a full grasp (left) and a reach task without grasp (right) executed by a hemi-parkinsonian rat in the SPRT.

The objective of this study is to characterize how midbrain dopaminergic degeneration can induce changes in M1 activity that can be visualized and quantified using *in vivo* calcium imaging in awake rats. To achieve this goal, we used single-photon fluorescent calcium imaging, a technique that combines the benefits of imaging and electrophysiological recording techniques to provide an assessment of both the systems and cellular responses in the disease and treatment states in a single analysis platform. We imaged calcium activity in the M1 of GCaMP6f-expressing rats as they transitioned from a naïve baseline to a 6-hydroxydopamine (6-OHDA)-lesioned hemi-parkinsonian state and then to a levodopa-treated state over the course of three months. Motor function and M1 calcium activity were chronically evaluated in the single pellet-reaching test (SPRT) (Fig. 1a). Here, we demonstrate that levodopa treatment of 6-OHDA-lesioned rats not only improves fine motor abilities but modulates M1 neuronal calcium activity in a manner dependent of the levels of dopaminergic lesion.

## Results

### Confirmation of GCaMP6f expression and PRISM lens placement

**Figure 2:**
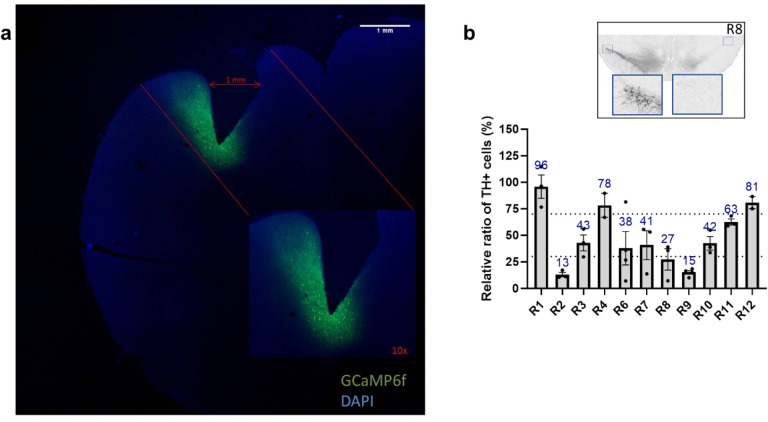
Histological assessment of fluorescent calcium indicator expression and PRISM lens implantation. **(a)** Representative image of GCaMP6f expression in M1 neurons of R3 relative to PRISM lens implantation site. **(b)**Histological quantification of TH+ neurons in the Substantia Nigra *pars compacta* (SNc) with representative midbrain image of R8.

Calcium activity data from seven of 13 rats were included in the analysis following both verification of PRISM lens placement in M1 and GCaMP6f expression (Fig. 2a). Of the six rats excluded, one rat (R5) was removed from the analysis because the baseplate detached from the headcap precluding microscope connection. An additional five rats (R9 – R13) were excluded because calcium signals were not detected in one or more imaging sessions. Histological analysis of GCaMP6f expression and PRISM lens localization showed that four of these rats had the PRISM lens misplaced relative to the GCaMP6f-expressing area, and one rat had extensive inflammatory reaction with cortical damage that hindered proper calcium imaging (see Table 1 for details).

### Characterization of Dopaminergic Lesion

Comparison of TH + neurons in the SNc of the 6-OHDA-lesioned hemisphere (right side) to TH + neurons on the non-lesioned control hemisphere (left side) allowed classification of rats as (i) high lesion level if TH + neuron loss was above 70%, (ii) mild lesion level if TH + neuron loss was between 30–69%, and (iii) low lesion level if TH + neuron loss was 0 to 29%. Three rats (R2, R8, R9) presented high lesion level, five rats (R3, R6, R7, R10, R11) presented mild lesion level, and three rats (R1, R4, R12) presented low lesion level (Fig. 2b).

### Levodopa improves fine motor abilities only in high lesion level hemi-parkinsonian rats

Following SPRT training, a set of 3–5 test sessions with 25 trials each was performed. For each session, M1 activity was recorded while rats extended either their left or right forelimb through the grid fence and grasped a sucrose pellet placed in the receptacle located just outside the behavioral setup. Their motor performance was recorded on video and synchronized to the calcium recordings. Categorization and quantification of fine motor movements was conducted by a trained investigator who was blinded to the treatment (Fig. 1b, **Supplementary Table 1**).

**Figure 3:**
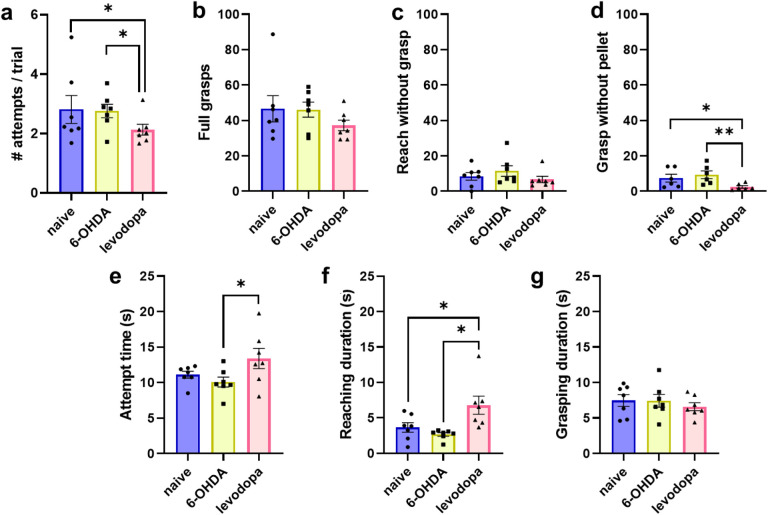
Levodopa improves fine motor abilities in hemi-parkinsonian rats. Rats performed better in the single pellet reaching test (SPRT) after levodopa treatment. Repeated measures two-way ANOVA followed by Tukey’s post-hoc test. Data are represented as mean ± SEM. N = 7, * p < 0.05; ** p < 0.01

Levodopa treatment improved overall performance in the SPRT compared to both naive (p = 0.03) and 6-OHDA-lesioned (p = 0.02) states by reducing the number of attempts executed to successfully reach and grasp the pellet in each trial (Fig. 3a). Interestingly, movements executed when a sucrose pellet was placed in the receptacle (full grasp and reach without grasp) were not affected (Figs. 3b and 3c). However, levodopa treatment resulted in a significant decrease in grasp movements when a sucrose pellet was not in the receptacle relative to naive and 6-OHDA-lesion, respectively (p = 0.028, p = 0.005) (Fig. 3d). This suggests that levodopa improves voluntary movements contributing to improved performance in the SPRT.

Additionally, the levodopa treatment state had an increased total attempt duration compared to the 6-OHDA lesion (Fig. 3e). We divided the total attempt duration into reaching duration (i.e., time to reach and grasp the sucrose pellet) and grasping duration (i.e., time during which grasp is maintained to bring the pellet to the animal’s mouth). Levodopa treatment increased the reaching duration relative to naïve and 6-OHDA-lesion (p = 0.029, p = 0.015), respectively (Fig. 3f). However, levodopa treatment did not affect grasping duration (Fig. 3g). This suggests an improved motor coordination and movement control of forelimbs and paws during the levodopa treatment state. Indeed, during the 6-OHDA-lesioned state, rats exhibited frequent shorter and incomplete ballistic paw/forelimb movements that did not fit the criteria for a full extension to reach or grasp a pellet beyond the grid fence.

**Figure 4:**
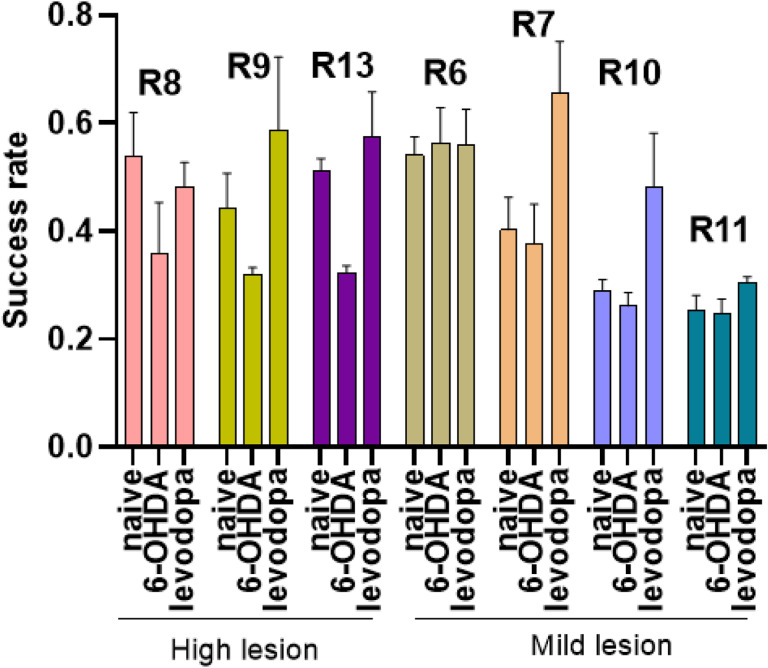
Success rate across naïve, 6-OHDA-lesioned, and levodopa-treated states. Rats with high lesion level presented worse success rate following 6-OHDA lesion. The success rate of these rats improved with levodopa treatment. Rats with a mild lesion did not show a similar pattern of changes in the success rate across the different states.

Motor task performance can be directly influenced by dopaminergic transmission.^31–33^ Thus, unilateral 6-OHDA lesioning of the nigrostriatal pathway should result in significant motor deficits evident in the SPRT. However, our results did not show significant motor deficits in fine motor function of rats in the 6-OHDA-lesioned state (Fig. 3). The success rate, defined as the number of successful attempts in which the rat grasped and ate a sucrose pellet divided by the total number of attempts, was used to evaluate the effect of dopaminergic lesion on motor behavior. Rats with high loss of TH + neurons (R8, R9, R13) showed a decreased success rate following 6-OHDA lesion followed by improvement after levodopa treatment. This pattern was not observed in rats with mild lesions (R6, R7, R10, R11) (Fig. 4).

Importantly, rats 1 through 5 received two days of training during the naïve state and a total of 15 trials per session (Table 1). These rats did not receive sufficient SPRT training during naïve state, which could bias comparisons in subsequent states,^7,34^ and were thus analyzed separately. For these rats (R1 – R5), we observed reduced performance in the SPRT following 6-OHDA lesion as shown by an increased number of attempts per trial and full grasps, and no improvement during levodopa treatment (**Supplementary Figure S1a, Supplementary Table S2**). Additionally, the success rate for rats 1–5 does not follow a similar pattern to rats 6–13 (Supplementary Figure S1b). Specifically, rat 2 does not show a clear pattern of reduced success rate during the 6-OHDA lesioned state, as expected from rats with high levels of dopaminergic neuronal loss.

### Changes in M1 calcium activity in response to dopaminergic lesion and levodopa treatment

**Figure 5:**
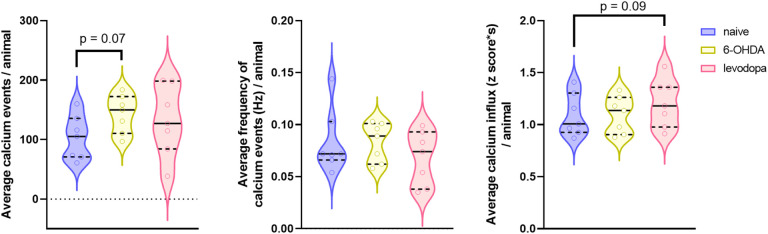
Neuronal calcium activity in the motor cortex of rats. Single-photon calcium recordings were acquired during naïve, 6-OHDA-lesion, and levodopa treatment states. Unilateral injection of 6-OHDA in the MFB and subsequent levodopa treatment produced changes in the M1 calcium activity. Data were analyzed using a linear mixed model with a random effect for animal. The average for each rat is displayed as median and interquartile range with the shape of the violin plots representing the distribution of individual data points. N = 7 rats (ranging within an average of 91 neurons recorded during naïve, 81 after 6-OHDA lesion, and 90 during levodopa treatment).

We recorded calcium signals from M1 neurons using a miniature head-mounted microscope while rats executed the SPRT. We assessed global changes in calcium activity by determining the total number of calcium events, the frequency of calcium events (Hz), and the magnitude of calcium influx using the average of the area under the curve (AUC) of each event. We obtained consistent calcium signals in all three states for seven rats (Table 1). We successfully maintained focal planes and tracked neuronal ensembles for 3–4 days within each state. However, we were unable to track the same neuronal ensembles across the three states due to intrinsic technical limitations of the device and surgical approach that creates slight shifts in the field of view over a prolonged period of time (> 2 weeks). Analysis of calcium activity showed that the 6-OHDA lesion was associated with an increase in the number of calcium events in M1 (p = 0.07) but had no effects on the frequency (p = 0.992) or magnitude (p = 0.948) of calcium events relative to naïve state. In contrast, levodopa treatment was not associated with changes in the total number of calcium events relative to naïve or 6-OHDA-lesion (p = 0.185, p = 0.563), respectively. Similarly, levodopa treatment was not associated with calcium frequency relative to naïve or 6-OHDA-lesion (p = 0.222, p = 0.225), respectively. However, it was associated with an increase in calcium influx magnitude relative to naïve and 6-OHDA-lesion (p = 0.090, p = 0.101), respectively (Fig. 5).

**Figure 6:**
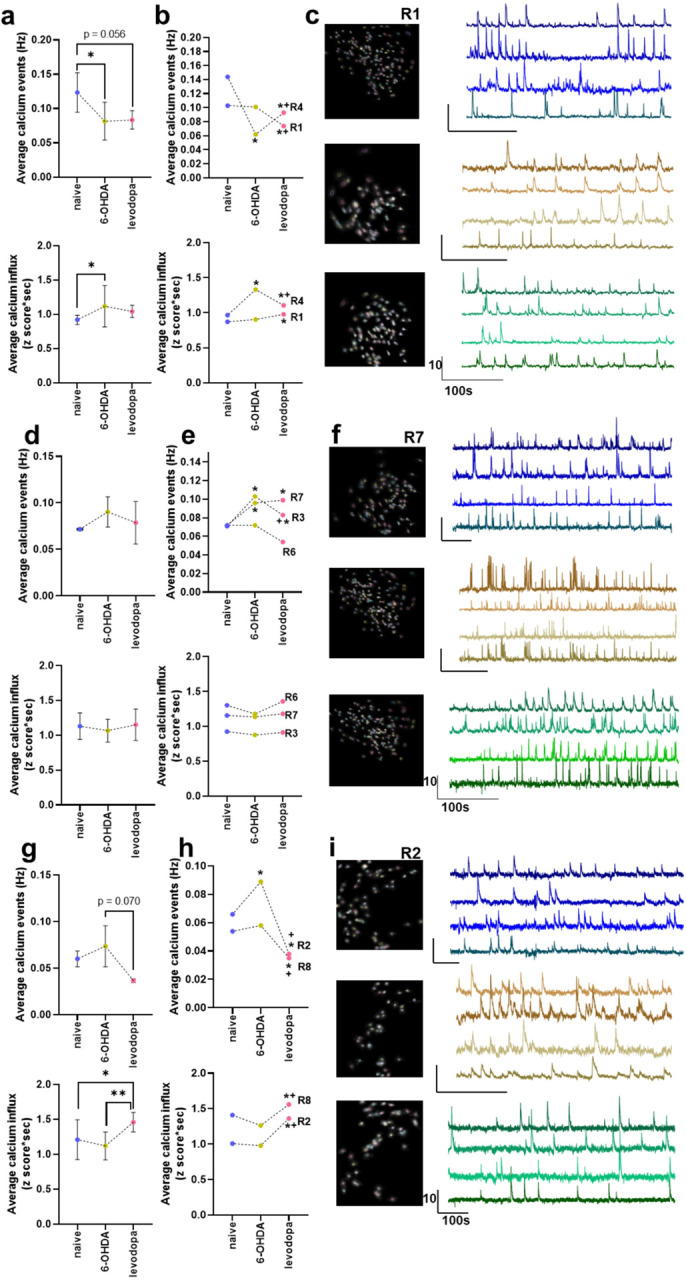
Neuronal calcium activity in the M1 of rats changes as a function of the level of dopaminergic lesion. M1 activity in rats with high level (>70 %) of dopaminergic lesion is more responsive to levodopa treatment whereas rats with low level of dopaminergic lesion (< 30 %) show changes in M1 activity after 6-OHDA lesion that are not influenced by levodopa. Data in **(a)**, **(d)**, and **(g)** are analyzed with mixed model analysis with random factors considering the means of lesion levels and are represented as median and interquartile range. Data in **(b)**, **(e)**, and **(h)** are analyzed with two-way ANOVA and are represented as mean. Representative images and calcium traces of rats with low lesion (R1) **(c)**, mild lesion (R7) **(f)**, and high lesion (R2) levels **(i)** at naïve, 6-OHDA-lesioned state, and levodopa-treated state. N = 7 rats ranging within an average of 91 neurons recorded during naïve, 81 after 6-OHDA lesion, and 90 during levodopa treatment. * p < 0.05, ** p < 0.01, + p < 0.05 compared to 6-OHDA lesion state.

We further assessed calcium activity in rats with high (R2, R8), mild (R3, R6, R7), and low (R1, R4) lesion levels to determine if SNc dopaminergic transmission influences neuronal activity in M1. The frequency of calcium events was significantly reduced for rats with low dopaminergic lesion levels during the 6-OHDA-lesioned state (p = 0.046) and the levodopa treated state (p = 0.056), the magnitude of calcium influx increased in the 6-OHDA-lesioned state (p = 0.038) with no changes in the levodopa treated state relative to naïve and 6-OHDA-lesion (p = 0.164, p = 0.367), respectively (Fig. 6a). The calcium events in R4 showed a significant reduction in frequency (p < 0.0001) and an increase in the magnitude of calcium influx in the 6-OHDA-lesioned state (p < 0.0001) followed by improvement of calcium event frequency (p = 0.0002) and calcium influx (p < 0.0001) with levodopa treatment. In contrast, no effects were observed in the 6-OHDA-lesioned state in R1 (calcium frequency p = 0.9675, calcium influx p = 0.7716) (Fig. 6b). No significant effects in the frequency and magnitude of calcium events were observed in rats with mild lesions during the 6-OHDA-lesion and levodopa treatment states (Fig. 6d). Rats with a mild lesion level (R3 and R7) showed increased calcium event frequency (p < 0.0001). However, levodopa treatment only improved calcium event frequency in R3 (p = 0.0004). These rats showed no changes in calcium influx magnitude (Fig. 6e). Levodopa treatment showed a reduction in frequency of calcium events in rats with high lesion levels compared to 6-OHDA-lesioned state (p = 0.07) and significantly increases in calcium influx magnitude compared to naïve (p = 0.014) and 6-OHDA-lesioned (p = 0.003) states (Fig. 6g). Rats with high lesion levels exhibited different responses: R2 showed increased calcium event frequency in the 6-OHDA-lesioned state (p < 0.0001) that was recovered by levodopa treatment (p < 0.0001) and increased calcium influx after levodopa treatment (p < 0.0001). In contrast, Rat 8 did not show significant changes in calcium event frequency (p = 0.7544) or calcium influx magnitude (p = 0.1428) in the 6-OHDA-lesioned state but showed decreased calcium event frequency (p < 0.0001) and increased calcium influx magnitude (p < 0.0001) in the levodopa treatment state (Fig. 6h).

**Figure 7:**
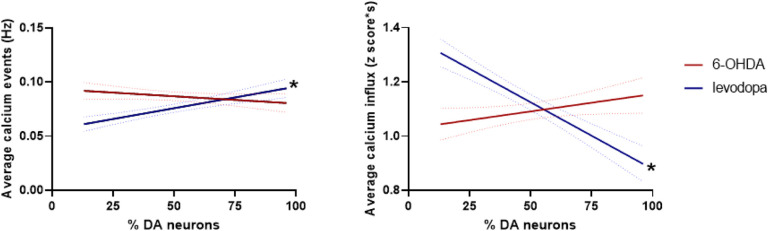
Levodopa modulates neuronal calcium activity in the M1 as a function of dopaminergic lesion. M1 calcium event frequency and calcium influx magnitude are not significantly affected by a gradual and steady loss of dopaminergic neurons as represented in the 6-OHDA lesion state. However, upon levodopa treatment, M1 calcium event frequency and influx magnitude are differentially modulated by the level of dopaminergic lesion. Simple linear regression, * p < 0.0001.

We confirmed the influence of dopaminergic transmission on M1 neurons by fitting the frequency and magnitude of calcium events and percentage of remaining SNc dopaminergic neurons using a simple linear regression model. The model showed that the frequency of calcium events in M1 was not affected by the gradual and steady loss of dopaminergic neurons in SNc during the 6-OHDA-lesioned state (F(1,571) = 2.40), p = 0.122). Instead, the model showed only a trend to decrease calcium influx magnitude (F(1,571) = 3.77), p = 0.053). However, when dopaminergic transmission is replenished with levodopa treatment, the frequency of calcium events in M1 decreases (F(1,633) = 24.54, p < 0.0001) while the influx magnitude of such events increases (F(1,633) = 63.24, p < 0.0001) with the loss of dopaminergic neurons (Fig. 7).

## Discussion

M1 excitability is modulated by dopaminergic projections from the VTA and by the net effect of the direct and indirect pathways of the cortico-basal ganglia-thalamic circuitry.^10,11^ In PD, dysfunctional dopaminergic signaling leads to an imbalance in the direct and indirect pathways which would result in exacerbated inhibitory input to M1 which is thought to be responsible for motor deficits.^15–17^ However, multiple animal model and clinical studies report conflicting results, showing increased,^5,18,19,25,26,35–38^ decreased,^29,39–44^ or unchanged^27,28,34,38^ activation of M1. Here, we propose that such inconsistencies may result from studying M1 excitability at different levels of dopaminergic loss. An initial analysis of M1 activity shows subtle trends towards M1 overexcitation following 6-OHDA lesion and levodopa treatment (Fig. 5). However, when we consider the level of midbrain dopaminergic depletion achieved with 6-OHDA lesion, we identify distinct patterns of M1 activation. Specifically, rats with less than 70% of dopaminergic depletion (low lesion level, Fig. 6a–c) showed decreased frequency and increased magnitude of calcium events with no significant effects in response to levodopa treatment. In contrast, rats with more than 30% of dopaminergic depletion (high lesion level, Fig. 6g–i) showed reduced frequency and significant increase in the magnitude of calcium events during levodopa treatment, with no significant changes during 6-OHDA-lesioned state. Additionally, rats with mild (30–69%) dopaminergic depletion (Fig. 6d–f) did not show changes in M1 activity. Importantly, it is worth noting that linear regression analysis reveals that the frequency of calcium events in the M1 tends to decrease while calcium influx magnitude tends to increase as the dopaminergic lesion level increases when rats are treated with levodopa but remain unaffected during the 6-OHDA lesion state, i.e., in the absence of treatment (Fig. 7).

D1R and D2R may have similar effects in M1 depending on the cortical layers and neuronal types in which they are expressed, as well as the anatomical origin of dopaminergic depletion.^7,9,45^ However, their overall signaling effect through cortico-basal ganglia-thalamic circuitry may have opposing effects. In our study, the meso-cortical dopaminergic pathway and cortico-basal ganglia-thalamic circuitry were not severely damaged in rats with low dopaminergic depletion, but the latter might still have minor dysfunction given that direct 6-OHDA administration into the MFB would affect dopaminergic projections primarily in the nigrostriatal pathway.^46,47^ Thus, subtle imbalances in the indirect pathway could result in increased M1 inhibition or no significant changes since dopaminergic meso-cortical projections might sustain M1 activity.^48,49^ If dopaminergic transmission is supplemented with levodopa, significant changes would not be observed likely because dopaminergic signaling would not be sufficiently imbalanced by the lesion. On the other hand, in high dopaminergic lesion, severe damage to the cortico-basal ganglia-thalamic circuitry would result in decreased M1 activity^15–17,50^ while severe damage to the meso-cortical pathway would result in increased M1 activity.^48,49^ Thus, the net effect would be no noticeable changes in M1 activity. However, levodopa treatment would restore deficient dopaminergic transmission and allow for recovery of M1 activity. Interestingly, studies assessing M1 activity changes induced by deep brain stimulation (DBS) of the subthalamic nucleus (STN) report decrease and regularization in M1 firing rate with stimulation,^51^ likely due to increased cortical GABAergic interneuron excitability^52–54^. The efficacy of both levodopa and STN DBS to alleviate parkinsonian symptoms in experimental animals and PD patients while modulating M1 activity reiterates the essential role of M1 in PD pathogenesis and supports the multi-circuitry regulation of M1 activity. Future experiments evaluating changes in M1 activity in response to STN DBS are necessary to further elucidate potential mechanisms of M1 activity modulation.

Impaired motor performance and/or bradykinesia were observed in high lesion level rats. However, mild lesion level rats did not show fine motor impairment in the SPRT. This reinforces the notion that motor deficits in PD are observed when more than 70% of nigral dopaminergic neurons are lost and a fine balance of the direct and indirect cortico-basal ganglia-thalamic pathways is no longer achieved.^55^ In contrast to Metz *et al*. (2001), who did not detect motor improvement with chronic levodopa treatment,^56^ we report a significant improvement in the performance of SPRT in the levodopa-treated state compared to the lesioned state with reduced number of attempts to successfully grasp and eat a pellet. Additionally, the attempt duration to reach a pellet increased after levodopa treatment, particularly when the pellet was present in the receptacle. In agreement with our findings pertaining grasping duration, Hyland and colleagues (2019) showed no changes in the terminal part of the reaches possibly because this is the deceleration part of the movement when velocities in lesioned rats and controls may converge.^34,57^ This suggests that levodopa-treated animals developed a motor control strategy focused on goal achievement with fine tuning of proximal and distal movements to accurately reach and grasp the pellet.

Limitations of our study include the fact that not every rat included in the calcium imaging analysis was included in the behavioral analysis. Therefore, we were unable to correlate M1 calcium activity with specific limb movements and draw spatiotemporal maps to evaluate neuronal ensembles encoding for specific movements. Such information would further elucidate the role of dopamine in M1 neuronal modulation to execute specific fine movements. Furthermore, it must be acknowledged that calcium imaging measures neuronal activity through the influx of calcium into neurons,^58^ which does not fully correspond to action potentials. Additionally, fluorescent calcium imaging relies on signal emitted by active cells. It is possible that neurons become quiescent in the 6-OHDA lesioned state and that calcium imaging picks up the signals only from neurons that remain active. This would result in the study of active neurons only, neglecting the population of inactive neurons, which may not be representative of the entire M1 neuronal population. Future studies seeking to correlate neuronal calcium signaling and action potentials would enhance the accuracy and significance of calcium imaging analysis.

In summary, our study demonstrates the feasibility of long-term (over three months) monitoring of M1 neuronal activity via calcium signaling and simultaneous motor behavior in awake, freely behaving hemi-parkinsonian rats and contributes to clarify the effects of disrupted midbrain dopaminergic transmission in M1. Furthermore, we demonstrate that while motor behavior is affected by severe changes in dopaminergic transmission, calcium activity in M1 is more susceptible to changes in response to quick fluctuations in dopaminergic transmission, rather than to a stabilized level of dopaminergic transmission with a higher or lower amplitude.

## Methods

### Animals

We used a total of 13 adult (8–9 weeks old) Sprague Dawley rats, including 8 males and 5 females, with an approximate weight of 250–280 g. The rats were kept on a standard 12-hour light/dark cycle in single housing at a constant 21°C temperature and 45% humidity with ad libitum access to water and food. After approval by the Mayo Clinic Institutional Animal Care and Use Committee (IACUC), all animal procedures and experiments were conducted following the terms and guidelines of the National Institutes of Health for the use of animals and complied with the ARRIVE guidelines.

### General Surgical Procedures

Anesthesia was induced using 5% isoflurane and maintained using 1–2% isoflurane via a nose cone. Anesthetized rats were placed on heated stage (37°C) and the skull was secured with blunt ear bars and a nose clamp incisor bar in a Kopf stereotaxic frame (Kopf Systems, Tujunga, USA). Analgesia was provided pre-operatively with a 1.0 mg/kg buprenorphine extended-release injection.

### Viral Transduction Procedure

On day one, the rat’s head was shaved and disinfected with betadine, and a midline scalp 1.5–2 cm incision was performed starting between the eyes and extending caudally to the level of the ears. The periosteum was removed, and the skull cleaned with 0.9% saline and sterile cotton swabs. A 0.5 mm burr hole was made above the M1 (AP + 2.5 mm, ML + 2.6 mm from bregma, DV −1.8 mm from dura mater). Coordinates for all brain regions targeted in the surgical procedures were determined using a rat brain atlas.^59^ The dura mater was incised to allow insertion of a 28-gauge needle and infusion of 500 nL of 110 vg/mL genetically encoded calcium indicator pENN.AAV9-CaMKII-GcaMP6f-WPRE-SV40 (Addgene Inc, Watertown, USA) at a rate of 100 nL/min. The needle was maintained in place for 5 min to prevent reflux and then withdrawn at a rate of 10 μm/s. The burr hole was covered with bone wax (Medtronic, Minneapolis, USA) and the skin incision was closed in one layer with simple interrupted stitches using 4 − 0 vicryl sutures. Rats were continuously monitored in a heated cage and returned to their home cage once they became ambulatory.

### GRIN Lens Implantation Procedure

On day 14, rats were implanted with a guide-cannula over the MFB and a GRIN lens was implanted in M1. Anesthesia, analgesia, and surgical approach were identical to the those described for the viral transfection procedure. The burr hole above M1 was widened to approximately 1.5 mm and a 1 mm dorsoventral incision was made in the cortex with a straight-edge #11 dissection knife attached to a stereotactic arm perpendicular to the brain surface to insert a 1 mm GRIN prism lens (Inscopix, Palo Alto, CA) into M1. The exposed areas surrounding the lens were covered with a biocompatible silicone elastomer Kwik-Sil (World Precision Instruments, Sarasota, FL). A second burr-hole (1.5 mm) was drilled to insert a 22G guide-cannula 2.0 mm above the MFB. Two skull screws were placed bilaterally anterior to bregma and two additional screws were placed posterior to bregma on the contralateral side of the lens and guide-cannula to secure a headcap built with Metabond quick adhesive (Parkell, Edgewood, US).

### Baseplating Procedure

On day 21, a baseplate (~ 0.5 g) was attached above the GRIN lens to allow for attachment and support of a miniature microscope. Rats were anesthetized and secured on the stereotaxic frame as described previously, but no analgesia was provided since this is a non-invasive procedure. isoflurane concentration was modulated to allow for visualization of calcium activity from M1 neurons via the GRIN lens to ensure correct placement of the baseplate as the baseplate was moved along three degrees of freedom (ML, AP, DV) in order to identify the location with maximal fluorescence. Once this location was identified, the baseplate was secured in place using Metabond.

### Food Restriction

Rats were put on a food restricted diet of 10–15 g standard food chow in order to entice them to grasp and eat sucrose pellets during the SPRT. Food restriction started 3 days prior to the first training session and was maintained during testing for a maximum of 3 weeks or until rats reached 90% of their initial body weight. Rats were weighed daily during the food restriction period.

### Chamber for Assessment of Motor Function

Motor function was assessed via SPRT using a custom built 360 × 80 mm chamber with transparent plastic sidewalls and a metal grid fence on one end, across which a plastic platform (88 × 45 × 25 cm) with receptacles for sucrose pellets was located just outside the fence. The fence openings were just wide enough for the rat to thrust out its forelimbs and grasp a single 45 mg sucrose pellet.

### SPRT Assessments of Motor Function

#### Habituation and Training Sessions

Training sessions started 5–7 days following baseplate implantation. One day before training, rats were habituated to the testing chamber for 5 min while receiving sucrose pellets. During the training sessions, rats were gently guided to reach through the fence and grasp sucrose pellets placed on the platform receptacle. The SPRT consisted of 25 trials in which the rat was allowed to grasp and eat a total of 25 sucrose pellets or until the time limit of 15 min expired. Each trial started with the placement of a sucrose pellet in the platform receptacle and ended after the rat ate the pellet. If the rat pushed the pellet beyond its reach, a new sucrose pellet was placed in the platform and was considered the same trial. Rats were connected to a dummy miniature microscope during the training sessions to habituate the animals to the weight and size of the microscope used during testing.

#### Testing Sessions

Test sessions began once rats were able to successfully grasp and eat 25 pellets within 15 minutes (usually after 3 to 5 days of training). Testing was conducted for 3 to 5 consecutive days, where rats were connected to a skull-mounted miniature microscope to record calcium activity from M1. Synchronized calcium activity and pellet reach behavioral data were acquired during test sessions.

The number of attempts in a given trial to successfully grasp and eat a sucrose pellet was not limited (Fig. 1B). An investigator blinded to the treatment analyzed and quantified the rats’ forelimb movements according to the following criteria:

- *full grasp*: extension of paw beyond the fence and grasping of a pellet. The success ratio was calculated based on categorization of full grasps in successful if the rat ate the pellet or failure if the rat did not eat or dropped the pellet after grasping it.
- *reach without grasp*: extension of the paw beyond the fence allowing to touch the pellet but not grasping it.
- *grasp without pellet*: extension of paw beyond the fence to attempt to grasp a pellet in the absence of a sucrose pellet in the receptacle.
- *attempt duration*: starts when paw lifts from the floor, extends beyond the fence, and ends when pellet is brought to mouth (full grasp) or when paw is placed back on the floor (reach without grasp).
- *reaching duration*: starts when paw lifts from the floor, extends beyond the fence, and ends when the pellet is grasped.
- *grasping duration:* starts when pellet is grasped and ends when pellet is put in mouth or dropped.

### 6-OHDA lesion and levodopa treatment

Rats were unilaterally infused with 3 μl of 6-OHDA hydrobromide (4 μg/μl solution of 0.02% sterile ascorbate saline) at a rate of 0.5 μl/min with 5 min for diffusion into the MFB the day after SPRT test sessions in the naïve state. To prevent 6-OHDA uptake by noradrenergic neurons, desipramine hydrochloride (25 mg/kg i.p.) was administered 30 min before 6-OHDA infusion. A period of 3 weeks was allowed following 6-OHDA lesioning for dopaminergic lesion stabilization. After stabilization, rats received a daily 10 mg/mL levodopa (Sigma Millipore, Burlington, MA, CAG1361009) intraperitoneal (i.p.) injection co-administered with a 1.25 mg/mL carbidopa decarboxylase inhibitor (Sigma Millipore, Burlington, MA, CAG1095506) dissolved in 0.9% saline for 14 days to restore dopaminergic levels while preventing peripheral levodopa degradation and increasing central concentration. SPRT habituation and training was initiated after 3–5 days of levodopa/carbidopa treatment.

### Histology

At the end of all experiments, rats received an i.p. overdose of pentobarbital sodium (100 mg/kg) and were transcardially perfused with 0.1 M phosphate buffered saline (PBS) followed by 4% paraformaldehyde. Rats were decapitated, their scalp exposed, skull opened, and their brains extracted and fixed overnight in 4% paraformaldehyde. Brains were stored in 30% glycerol. Cryosectioning with 40 μm slices was performed on a sliding microtome (Leica Biosystems, Wetzlar, Germany). Tissue sections were stored in 0.1% Sodium Azide in 0.1 M PBS.

Coronal M1 slices were mounted onto glass slides and coverslipped with VectaShield containing DAPI (Vector Laboratories, Newark, CA) to assess PRISM lens placement and GCaMP6f expression. If the location of the PRISM lens or GCaMP6f expression were not in the M1 region, the corresponding rats were excluded from the analysis. To quantify dopaminergic cell loss, we performed immunohistochemical staining using tyrosine-hydroxylase (TH). Coronal slices of the SNc were rinsed in 0.01 M PBS with 0.2% Triton X-100 (PBS-Tx), incubated in 3% H_2_O_2_ for 10 min to quench endogenous peroxidase activity and blocked in 10% normal goat serum (NGS) in PBS-Tx for 1 h. Slices were incubated with the primary antibody, anti-TH (rabbit polyclonal, Abcam 1:4000), overnight at 4° C on a shaker. Following primary antibody incubation, sections were rinsed in PBS-Tx and incubated in 10% NGS with the biotinylated goat anti-rabbit IgG secondary antibody (Vector Laboratories, 1:500) for 1 h at room temperature. Following the rinse in PBS-Tx, slices were incubated with an avidin-biotin enzyme complex (VECTASTAIN^®^ Elite^®^ ABC-HRP kit, PK-6101, Vector Laboratories), rinsed in 0.1 M PBS, and incubated with 3,3’-Diaminobenzidine chromogen (DAB Substrate kit, SK-4100, Vector Laboratories). After final rinse in 0.1 M PBS, slices were mounted onto glass slides and coverslipped with mounting media (Eukitt^®^ Quick-hardening mounting medium, 03989, Sigma-Aldrich). Slides were imaged under a microscope (Keyence, BZ-X800).

### Characterization of Dopaminergic Lesion

Dopaminergic neuron loss was assessed by bilateral quantification of TH-positive neurons in the SNc. Two to four SNc-containing midbrain sections from each animal were evaluated and the relative ratio of TH + cells was determined as the percentage of TH + cells in the lesioned SNc relative to the intact SNc. Midbrain sections of R13 were damaged and densitometry of TH + terminals was conducted to evaluate the level of dopaminergic depletion for this animal (**Supplementary Figure S2**). The number of TH + neurons in the SNc of the 6-OHDA-lesioned hemisphere (right side) was compared to the number of TH + neurons on the non-lesioned control hemisphere (left side).

### Calcium data collection

Cortical calcium imaging was performed using an nVista miniature microscope (Inscopix, Palo Alto, CA) weighing approximately 2 g and attached to the baseplate on the rat’s head while rats moved freely in the SPRT chamber. Real-time imaging was transmitted to a computer via a data acquisition (DAQ) system connected to the miniature microscope. Each test session rendered one or more videos of calcium activity. Thus, each state (naïve, 6-OHDA-lesioned, and levodopa-treated) produced at least four videos for data analysis.

### Calcium data analysis

Calcium data for each state were compared and only the videos with the same field of view were stitched to allow uniform preprocessing and longitudinal registration of the neuroanatomy. Calcium data across states were not processed longitudinally due to differences in field of view. Each calcium data set was preprocessed by spatially down sampling each video (by a factor of two), cropping the field of view to the region with increased calcium signals, and motion correcting using the Turboreg algorithm implemented in the Inscopix Data Processing Software (IDPS) (Inscopix, Palo Alto, CA). Data were then exported as ISXD files and loaded into MATLAB (The MathWorks, Natick, MA) to extract putative neurons or regions of interest (ROIs) using a constrained non-negative matrix factorization (CNMF-E) algorithm.^60^ The ring model of background fluorescence was used for all data sets. Acceptance parameters were selected based on the data and included neuron diameter ≥ 7px, signal-to-noise ratio (SNR) between 3 and 5. Next, the CNMF-E-detected ROIs were loaded into IDPS for visual inspection of the shape and traces of each ROI and additional inclusion criteria were applied by the observer to ensure that (i) a single ROI outlined only one neuron, (ii) a single neuron was not labeled by multiple ROIs, and (iii) selected ROIs were present in all videos within each state. ROIs that didn’t meet these criteria were excluded from further analysis.

A peak detection algorithm was used to extract individual calcium events from raw calcium intensity traces for each ROI in MATLAB. Additionally, the area under curve (AUC) was calculated to measure the total calcium influx using the event onset and offset of each event, indicating the activity level for each specific neuron. Calcium intensity for selected ROIs was converted to a z-score scale to allow comparisons between rats across states. The calcium event rate was calculated using Eq. (1). The average calcium influx was determined by Eq. (2) in each state. Finally, event rate and average calcium influx data were used for statistical analysis and effect comparisons between states in GraphPad Prism.


(1) $$\frac{\text{total}\;\text{number}\;\text{of}\;\text{calciumevents}}{\text{total}\;\text{number}\;\text{of}\;\text{frames}}\times \text{frame}\;\text{rate}\;(20\text{Hz})$$

(2) $$\frac{\Sigma \text{AUC}}{\text{total}\;\text{number}\;\text{of}\;\text{calciumevents}}$$

### Statistical Analysis

Statistical analysis of the SPRT was performed using a repeated measures two-way ANOVA with subject and state as the independent variables. Calcium imaging analysis was performed using a mixed-model effects to account for the different number of neurons recorded in each state. Tukey’s multiple comparisons test was used to assess significant differences between states in both behavioral and calcium activity analyses. Analysis of calcium activity for individual animals was performed using a two-way ANOVA followed by a Tukey’s multiple comparisons test to evaluate the effects of each state on M1 calcium activity. The effects of dopaminergic lesion on M1 calcium activity were further assessed by applying a simple linear regression model of the average of calcium event frequency and the average of calcium influx magnitude for each rat relative to their percentage of TH + neurons.

## Figures and Tables

**Table 1:** Description of histological assessment and reasons for removal from data analysis.

Subject ID	Histology	SPRT	Ca^2^+ activity	Reason for removal from SPRT or Ca^2^+ imaging analysis
R1	Low TH + loss	Yes: 15 trials	Yes	SPRT protocol different from final analysis
R2	High TH + loss	Yes: 15 trials	Yes	SPRT protocol different from final analysis
R3	Mild TH + loss	Yes: 15 trials	Yes	SPRT protocol different from final analysis
R4	Low TH + loss	Yes: 15 trials	Yes	SPRT protocol different from final analysis
R5	Not assessed	Yes: 15 trials	No	SPRT protocol different from final analysis; Baseplate detached from headcap
R6	Mild TH + loss	Yes: 25 trials	Yes	N/A
R7	Mild TH + loss	Yes: 25 trials	Yes	N/A
R8	High TH + loss	Yes: 25 trials	Yes	N/A
R9	High TH + loss	Yes: 25 trials	No	No calcium activity during levodopa treatment state
R10	Mild TH + loss	Yes: 25 trials	No	No calcium activity during 6-OHDA lesion and levodopa treatment states
R11	Mild TH + loss	Yes: 25 trials	No	No calcium activity during 6-OHDA lesion and levodopa treatment states
R12	Low TH + loss	No	No	No calcium activity in any state, inflammation in M1
R13	High TH + loss in the STR	Yes: 25 trials	No	No calcium activity during levodopa treatment state

## Data Availability

The datasets and codes used and/or analyzed during the current study are available from the corresponding authors on reasonable request.

## References

[1] Beitz J. M. Parkinson’s disease: a review. Front Biosci (Schol Ed) 6, 65–74, doi:10.2741/s415(2014). 24389262

[2] Capriotti T. & Terzakis K. Parkinson Disease. Home Healthc Now 34, 300–307, doi:10.1097/nhh.0000000000000398 (2016). 27243427

[3] Halli-Tierney A. D., Luker J. & Carroll D. G. Parkinson Disease. Am Fam Physician 102, 679–691 (2020). 33252908

[4] Underwood C. F. & Parr-Brownlie L. C. Primary motor cortex in Parkinson’s disease: Functional changes and opportunities for neurostimulation. Neurobiol Dis 147, 105159, doi:10.1016/j.nbd.2020.105159 (2021). 33152506

[5] Burciu R. G. & Vaillancourt D. E. Imaging of Motor Cortex Physiology in Parkinson’s Disease. Mov Disord 33, 1688–1699, doi:10.1002/mds.102 (2018). 30280416 PMC6261674

[6] Cousineau J., Plateau V., Baufreton J. & Le Bon-Jégo M. Dopaminergic modulation of primary motor cortex: From cellular and synaptic mechanisms underlying motor learning to cognitive symptoms in Parkinson’s disease. Neurobiol Dis 167, 105674, doi:10.1016/j.nbd.2022.105674 (2022). 35245676

[7] Molina-Luna K. Dopamine in motor cortex is necessary for skill learning and synaptic plasticity. PLoS One 4, e7082, doi:10.1371/journal.pone.0007082 (2009). 19759902 PMC2738964

[8] Vitrac C. & Benoit-Marand M. Monoaminergic Modulation of Motor Cortex Function. Front Neural Circuits 11, 72, doi:10.3389/fncir.2017.00072 (2017). 29062274 PMC5640772

[9] Cousineau J., Plateau V., Baufreton J. & Le Bon-Jégo M. Dopaminergic modulation of primary motor cortex: From cellular and synaptic mechanisms underlying motor learning to cognitive symptoms in Parkinson’s disease. Neurobiology of Disease 167, 105674, doi:10.1016/j.nbd.2022.105674 (2022). 35245676

[10] Hosp J. A., Nolan H. E. & Luft A. R. Topography and collateralization of dopaminergic projections to primary motor cortex in rats. Experimental Brain Research 233, 1365–1375, doi:10.1007/s00221-015-4211-2 (2015). 25633321

[11] Vitrac C. Dopamine control of pyramidal neuron activity in the primary motor cortex via D2 receptors. Frontiers in Neural Circuits 8, doi:10.3389/fncir.2014.00013 (2014). PMC3937764 24616667

[12] Lanciego J. L., Luquin N. & Obeso J. A. Functional neuroanatomy of the basal ganglia. Cold Spring Harb Perspect Med 2, a009621, doi:10.1101/cshperspect.a009621 (2012). 23071379 PMC3543080

[13] Fazl A. & Fleisher J. Anatomy, Physiology, and Clinical Syndromes of the Basal Ganglia: A Brief Review. Semin Pediatr Neurol 25, 2–9, doi:10.1016/j.spen.2017.12.005 (2018). PMC6039104 29735113

[14] Groenewegen H. J. The basal ganglia and motor control. Neural Plast 10, 107–120, doi:10.1155/np.2003.107 (2003). 14640312 PMC2565420

[15] DeLong M. & Wichmann T. Update on models of basal ganglia function and dysfunction. Parkinsonism Relat Disord 15 Suppl 3, S237–240, doi:10.1016/s1353-8020(09)70822-3 (2009). 20082999 PMC4275124

[16] Nambu A. Seven problems on the basal ganglia. Curr Opin Neurobiol 18, 595–604, doi:10.1016/j.conb.2008.11.001 (2008). 19081243

[17] Obeso J. A. The basal ganglia in Parkinson’s disease: current concepts and unexplained observations. Ann Neurol 64 Suppl 2, S30–46, doi:10.1002/ana.21481 (2008). 19127584

[18] Mure H. Parkinson’s disease tremor-related metabolic network: characterization, progression, and treatment effects. Neuroimage 54, 1244–1253, doi:10.1016/j.neuroimage.2010.09.028 (2011). 20851193 PMC2997135

[19] Rascol O. Cortical motor overactivation in parkinsonian patients with L-dopa-induced peak-dose dyskinesia. Brain 121 (Pt 3), 527–533, doi:10.1093/brain/121.3.527 (1998). 9549528

[20] Mohl B., Berman B. D., Shelton E. & Tanabe J. Levodopa response differs in Parkinson’s motor subtypes: A task-based effective connectivity study. J Comp Neurol 525, 2192–2201, doi:10.1002/cne.24197 (2017). 28256710 PMC6301039

[21] Buhmann C. Pharmacologically modulated fMRI–cortical responsiveness to levodopa in drug-naive hemiparkinsonian patients. Brain 126, 451–461, doi:10.1093/brain/awg033 (2003). 12538411

[22] Planetta P. J. Distinct functional and macrostructural brain changes in Parkinson’s disease and multiple system atrophy. Hum Brain Mapp 36, 1165–1179, doi:10.1002/hbm.22694 (2015). 25413603 PMC4950674

[23] Wu T. Effective connectivity of brain networks during self-initiated movement in Parkinson’s disease. Neuroimage 55, 204–215, doi:10.1016/j.neuroimage.2010.11.074 (2011). 21126588

[24] Helmich R. C. Spatial remapping of cortico-striatal connectivity in Parkinson’s disease. Cereb Cortex 20, 1175–1186, doi:10.1093/cercor/bhp178 (2010). 19710357

[25] Yu H., Sternad D., Corcos D. M. & Vaillancourt D. E. Role of hyperactive cerebellum and motor cortex in Parkinson’s disease. Neuroimage 35, 222–233, doi:10.1016/j.neuroimage.2006.11.047 (2007). 17223579 PMC1853309

[26] Sabatini U. Cortical motor reorganization in akinetic patients with Parkinson’s disease: a functional MRI study. Brain 123 (Pt 2), 394–403, doi:10.1093/brain/123.2.394 (2000). 10648446

[27] Goldberg J. A. Enhanced synchrony among primary motor cortex neurons in the 1-methyl-4-phenyl-1,2,3,6-tetrahydropyridine primate model of Parkinson’s disease. J Neurosci 22, 4639–4653, doi:10.1523/jneurosci.22-11-04639.2002 (2002). 12040070 PMC6758785

[28] Doudet D. J., Gross C., Arluison M. & Bioulac B. Modifications of precentral cortex discharge and EMG activity in monkeys with MPTP-induced lesions of DA nigral neurons. Exp Brain Res 80, 177–188, doi:10.1007/bf00228859 (1990). 1972680

[29] Pasquereau B. & Turner R. S. Primary motor cortex of the parkinsonian monkey: differential effects on the spontaneous activity of pyramidal tract-type neurons. Cereb Cortex 21, 1362–1378, doi:10.1093/cercor/bhq217 (2011). 21045003 PMC3097989

[30] Costa R. M. Rapid alterations in corticostriatal ensemble coordination during acute dopamine-dependent motor dysfunction. Neuron 52, 359–369, doi:10.1016/j.neuron.2006.07.030 (2006). 17046697

[31] Boschen S. L., Andreatini R. & da Cunha C. Activation of postsynaptic D2 dopamine receptors in the rat dorsolateral striatum prevents the amnestic effect of systemically administered neuroleptics. Behavioural Brain Research 281, 283–289, doi:10.1016/j.bbr.2014.12.040 (2015). 25546724

[32] Wietzikoski E. C. Roles of D1-like dopamine receptors in the nucleus accumbens and dorsolateral striatum in conditioned avoidance responses. Psychopharmacology 219, 159–169, doi:10.1007/s00213-011-2384-3 (2012). 21720753

[33] Boschen S. L., Wietzikoski E. C., Winn P. & Cunha C. D. The role of nucleus accumbens and dorsolateral striatal D2 receptors in active avoidance conditioning. Neurobiology of Learning and Memory 96, 254–262, doi:10.1016/j.nlm.2011.05.002 (2011). 21619938

[34] Hyland B. I., Seeger-Armbruster S., Smither R. A. & Parr-Brownlie L. C. Altered Recruitment of Motor Cortex Neuronal Activity During the Grasping Phase of Skilled Reaching in a Chronic Rat Model of Unilateral Parkinsonism. J Neurosci 39, 9660–9672, doi:10.1523/jneurosci.0720-19.2019 (2019). 31641050 PMC6880456

[35] Thobois S. Overactivation of primary motor cortex is asymmetrical in hemiparkinsonian patients. Neuroreport 11, 785–789, doi:10.1097/00001756-200003200-00026 (2000). 10757520

[36] Vercruysse S. The neural correlates of upper limb motor blocks in Parkinson’s disease and their relation to freezing of gait. Cereb Cortex 24, 3154–3166, doi:10.1093/cercor/bht170 (2014). 23861319

[37] Degos B., Deniau J.-M., Chavez M. & Maurice N. Subthalamic Nucleus High-Frequency Stimulation Restores Altered Electrophysiological Properties of Cortical Neurons in Parkinsonian Rat. PLOS ONE 8, e83608, doi:10.1371/journal.pone.0083608 (2014). PMC3877054 24391793

[38] Lee L. N. Deep brain stimulation rectifies the noisy cortex and irresponsive subthalamus to improve parkinsonian locomotor activities. NPJ Parkinsons Dis 8, 77, doi:10.1038/s41531-022-00343-6 (2022). 35725730 PMC9209473

[39] Berding G. Resting regional cerebral glucose metabolism in advanced Parkinson’s disease studied in the off and on conditions with [(18)F]FDG-PET. Mov Disord 16, 1014–1022, doi:10.1002/mds.1212 (2001). 11748732

[40] Hilker R. Subthalamic nucleus stimulation restores glucose metabolism in associative and limbic cortices and in cerebellum: evidence from a FDG-PET study in advanced Parkinson’s disease. J Cereb Blood Flow Metab 24, 7–16, doi:10.1097/01.wcb.0000092831.44769.09 (2004). 14688612

[41] Pasquereau B., DeLong M. R. & Turner R. S. Primary motor cortex of the parkinsonian monkey: altered encoding of active movement. Brain 139, 127–143, doi:10.1093/brain/awv312 (2016). 26490335 PMC4794619

[42] Rios A. Differential Changes in the Lateralized Activity of identified Projection Neurons of Motor Cortex in Hemiparkinsonian Rats. eNeuro 6, doi:10.1523/eneuro.0110-19.2019 (2019). PMC6620387 31235466

[43] Parr-Brownlie L. C. & Hyland B. I. Bradykinesia induced by dopamine D2 receptor blockade is associated with reduced motor cortex activity in the rat. J Neurosci 25, 5700–5709, doi:10.1523/jneurosci.0523-05.2005 (2005). 15958736 PMC6724886

[44] Li Q. Therapeutic deep brain stimulation in Parkinsonian rats directly influences motor cortex. Neuron 76, 1030–1041, doi:10.1016/j.neuron.2012.09.032 (2012). 23217750

[45] Rioult-Pedotti M.-S., Pekanovic A., Atiemo C. O., Marshall J. & Luft A. R. Dopamine Promotes Motor Cortex Plasticity and Motor Skill Learning via PLC Activation. PLOS ONE 10, e0124986, doi:10.1371/journal.pone.0124986 (2015). 25938462 PMC4418826

[46] Ferro M. M. Comparison of bilaterally 6-OHDA- and MPTP-lesioned rats as models of the early phase of Parkinson’s disease: Histological, neurochemical, motor and memory alterations. Journal of neuroscience methods 148, 78–87, doi:10.1016/j.jneumeth.2005.04.005 (2005). 15939479

[47] Cui J. Characterization of graded 6-Hydroxydopamine unilateral lesion in medial forebrain bundle of mice. Scientific reports 14, 3721, doi:10.1038/s41598-024-54066-0 (2024). 38355892 PMC10866897

[48] Cousineau J. Dopamine D2-Like Receptors Modulate Intrinsic Properties and Synaptic Transmission of Parvalbumin Interneurons in the Mouse Primary Motor Cortex. eNeuro 7, doi:10.1523/eneuro.0081-20.2020 (2020). PMC7240291 32321772

[49] Swanson O. K., Evinger C., Plotkin J., Role L. & Bishop C. Primary Motor Cortex Circuitry in a Mouse Model of Parkinson’s Disease, State University of New York at Stony Brook, (2020).

[50] McGregor M. M. & Nelson A. B. Circuit Mechanisms of Parkinson’s Disease. Neuron 101, 1042–1056, doi:10.1016/j.neuron.2019.03.004 (2019). 30897356

[51] Degos B., Deniau J. M., Chavez M. & Maurice N. Subthalamic nucleus high-frequency stimulation restores altered electrophysiological properties of cortical neurons in parkinsonian rat. PLoS One 8, e83608, doi:10.1371/journal.pone.0083608 (2013). 24391793 PMC3877054

[52] Valverde S. Deep brain stimulation-guided optogenetic rescue of parkinsonian symptoms. Nat Commun 11, 2388, doi:10.1038/s41467-020-16046-6 (2020). 32404907 PMC7220902

[53] Däuper J. Effects of subthalamic nucleus (STN) stimulation on motor cortex excitability. Neurology 59, 700–706, doi:10.1212/wnl.59.5.700 (2002). 12221160

[54] Pierantozzi M. Deep brain stimulation of both subthalamic nucleus and internal globus pallidus restores intracortical inhibition in Parkinson’s disease paralleling apomorphine effects: a paired magnetic stimulation study. Clin Neurophysiol 113, 108–113, doi:10.1016/s1388-2457(01)00694-0 (2002). 11801431

[55] Mazzoni P., Shabbott B. & Cortés J. C. Motor control abnormalities in Parkinson’s disease. Cold Spring Harb Perspect Med 2, a009282, doi:10.1101/cshperspect.a009282 (2012). 22675667 PMC3367543

[56] Metz G. A., Farr T., Ballermann M. & Whishaw I. Q. Chronic levodopa therapy does not improve skilled reach accuracy or reach range on a pasta matrix reaching task in 6-OHDA dopamine-depleted (hemi-Parkinson analogue) rats. Eur J Neurosci 14, 27–37, doi:10.1046/j.0953-816x.2001.01615.x (2001). 11488946

[57] Whishaw I. Q. Making two movements at once: impairments of movement, posture, and their integration underlie the adult skilled reaching de cit of neonatally dopamine-depleted rats. Behav Brain Res 61, 65–77, doi:10.1016/0166-4328(94)90009-4 (1994). 8031497

[58] Huang T. & Ye Q. In vivo calcium imaging and Parkinson’s disease. Sci China Life Sci 59, 1338–1340, doi:10.1007/s11427-016-0356-6 (2016). 27921233

[59] Paxinos G. & Watson C. The Rat Brain in Stereotaxic Coordinates. 7th Edition edn, (Academic Press, 2013).

[60] Zhou P. E cient and accurate extraction of in vivo calcium signals from microendoscopic video data. eLife 7, e28728, doi:10.7554/eLife.28728 (2018). 29469809 PMC5871355

